# Identifying sex differences in predictors of epicardial fat cell morphology

**DOI:** 10.1080/21623945.2022.2073854

**Published:** 2022-05-19

**Authors:** Helen M. M. Waddell, Matthew K. Moore, Morgan A. Herbert-Olsen, Martin K. Stiles, Rexson D. Tse, Sean Coffey, Regis R. Lamberts, Hamish M. Aitken-Buck

**Affiliations:** a Department of Physiology, HeartOtago, School of Biomedical Sciences, University of Otago, Dunedin, New Zealand; b Department of Medicine, HeartOtago, Dunedin School of Medicine, University of Otago, Dunedin, New Zealand; c Department of Cardiology, Waikato District Health Board, Hamilton, New Zealand; d Waikato Clinical School, University of Auckland, Hamilton, New Zealand; e Department of Forensic Pathology, LabPLUS, Auckland City Hospital, Auckland, New Zealand; f Department of Cardiology, Dunedin Hospital, Southern District Health Board, Dunedin, New Zealand

**Keywords:** Epicardial adipose tissue, sex differences, fat cell size, paracardial adipose tissue

## Abstract

Predictors of overall epicardial adipose tissue deposition have been found to vary between males and females. Whether similar sex differences exist in epicardial fat cell morphology is currently unknown. This study aimed to determine whether epicardial fat cell size is associated with different clinical measurements in males and females. Fat cell sizes were measured from epicardial, paracardial, and appendix adipose tissues of post-mortem cases (*N*= 118 total, 37 females). Epicardial, extra-pericardial, and visceral fat volumes were measured by computed tomography from a subset of cases (*N*= 70, 22 females). Correlation analyses and stepwise linear regression were performed to identify predictors of fat cell size in males and females. Median fat cell sizes in all depots did not differ between males and females. Body mass index (BMI) and age were independently predictive of epicardial, paracardial, and appendix fat cell sizes in males, but not in females. Epicardial and appendix fat cell sizes were associated with epicardial and visceral fat volumes, respectively, in males only. In females, paracardial fat cell size was associated with extra-pericardial fat volume, while appendix fat cell size was associated with BMI only. No predictors were associated with epicardial fat cell size in females at the univariable or multivariable levels. To conclude, no clinical measurements were useful surrogates of epicardial fat cell size in females, while BMI, age, and epicardial fat volume were independent, albeit weak, predictors in males only.

## Introduction

Epicardial adipose tissue (EAT) is the visceral fat depot located between the myocardium and the visceral serous pericardium [1]. In recent decades, interest in EAT has grown due to advances in non-invasive imaging of the fat depot and the strong clinical associations of EAT deposition with cardio-metabolic disease [2–4]. Pre-clinical investigation of EAT morphology exclusively uses biopsies from either surgical patients or post-mortem cases, due to the inaccessibility of EAT. As a result, there are gaps in the basic understanding of EAT and the fat cells comprising the tissue. Some evidence suggests that epicardial fat cell size may be altered in certain cardiac pathologies such as atrial fibrillation and coronary artery disease [5,6]. In addition, our recent studies indicate that epicardial fat cell morphology might share unique relationships with body mass index (BMI) and EAT thickness [7,8]. However, it remains unknown whether sex differences exist in epicardial fat cell morphology.

Sex differences in other adipose depots have been demonstrated previously [9]. For instance, when compared to males matched for BMI and age, females tend to accumulate more adipose in subcutaneous gluteal and femoral depots in a manner that results in females having larger fat cells in those areas [10–12]. Conversely, males tend to have greater central visceral adipose deposition and larger visceral omental fat cells than females [13,14]. Overall EAT deposition has previously been found to vary according to sex. Some studies show sex differences in absolute EAT volume or thickness [15,16], especially in elderly individuals [17], while others show that the utility of EAT deposition in predicting cardiovascular disease mortality or parameters of cardiac function differs between males and females [17–19]. Whether these sex differences in EAT deposition are paralleled by sex differences in epicardial fat cell morphology, as is found in other visceral depots, requires investigation.

Therefore, the current study aimed to identify predictors of epicardial fat cell size in a cohort of post-mortem cases, while also assessing whether sex influences the utility of different variables to predict epicardial fat cell size. We found that epicardial fat cell size, like fat cells from paracardial and visceral appendix origins, is associated with BMI, age, and EAT volume when analysed as a total cohort, and in male cases only. Importantly, no available variable outside of paracardial fat cell size was associated with epicardial fat cell size in females. These findings provide important clarification of epicardial fat cell size predictors while also highlighting the necessity to adjust for sex in cardiac adipose research.

## Methods

### Post-mortem cases

Adipose tissue biopsies and adipose deposition measurements were collected as part of a 6-month prospective study from consecutive cases undergoing routine coronial post-mortem examination between November 2019 and April 2021 at Auckland City Hospital, Auckland, New Zealand. All examinations were authorized by the Chief Coroner. Provided that no individuals could be identified by information provided by the forensic pathologist, ethical review by the University of Otago Human Ethics Committee was deemed not required for this study. No cases were paediatric or from a suspicious cause of death. Additionally, no cases were decomposed, severely malnourished, or cachectic on observation. Available from each post-mortem case was age, sex, and body weight and height, which was to calculate BMI (body weight [kg]/body height [m]^2^). Identifying information was available only to the forensic pathologist and no identifying information could be deduced from the adipose images or computed tomography (CT) scans used for analysis.

The post-mortem cases included in this study were analysed as a total case cohort (*N* = 118) from which fat cell size measurements were available. Additional analysis was performed on a subset of the total case cohort (*N* = 70) from which both fat cell size and fat volume measurements were available.

### Adipose tissue sampling and processing

Adipose was procured from post-mortem cases as previously described [7]. Briefly, a sample of EAT was taken between the right coronary artery orifice and the right lateral ventricular wall. A paracardial fat biopsy was taken from the routine examination of the pericardial wall. Visceral appendix fat was sampled as part of routine appendix examination. All adipose biopsies were fixed in 10% formalin for 24–48 hours before transfer to an accredited histological processing laboratory (LabPLUS, Auckland City Hospital). Fixed tissues were embedded in paraffin and sectioned at 4 µm before being stained by an automated processor (Leica Multistainer model ST5020, Leica Biosystems, Germany) with haematoxylin and eosin. Histological sections were visualized using an Olympus BX53 microscope (Olympus, Japan) using a UPlanFL N 20x objective. Images were captured using an Olympus UC50 digital camera (Olympus, Japan). Representative fat cell images for each fat depot from male and female cases are shown in **Figure S1**.

### Adipocyte size measurement

Adipocyte sizes were measured in the histological sections using Aperio ImageScope software (Leica Biosystems Pathology Imaging, Germany) as previously described [7]. Adipocytes were excluded from analysis if containing a broken cell membrane or if cut off by the image edge. All other adipocytes within the field of view were included in the analysis.

### Post-mortem computed tomography

For this study, a subset of post-mortem cases underwent a non-contrast CT scan (Siemens SOMATOM Scope, Healthineers AG, Germany) of the head and torso in a supine position prior to the post-mortem examination. Axial slices were used for reproducibility, and in all cases Hounsfield units between −190 and −30 were considered adipose tissue, as used previously to define adipose on non-contrast CT [20]. Measurements were made using 3D Slicer. EAT volume was measured as fat within the pericardial sac, from the superior tip of the right atrial appendage to the visualization of the right coronary artery running through the inferior atrioventricular groove inferiorly. Extra-pericardial adipose tissue was defined as the fat outside of the pericardium that was not lung tissue or mediastinal fat, at the same axial levels as EAT. Visceral fat volume was measured as fat within the abdominal cavity at one axial slice through the centre of the umbilicus. To reduce noise, median smoothing was used with a kernel size of 3 × 3 mm.

### Statistical analysis

Raw data are presented as median values ± interquartile range. Data normality was assessed by Shapiro–Wilk test. Differences in male and female post-mortem case characteristics and adipose measures were assessed using unpaired two-tailed *t*-tests or Mann–Whitney test based on normality of data distribution. Differences in fat cell sizes were assessed using Kruskal–Wallis tests followed by Dunn’s multiple comparison test or two-way ANOVA followed by Tukey’s multiple comparison test. Additional analysis of fat cell number was performed using qualitative assessment of size frequency distribution of all fat cells measured, as well as comparison of median and lower and upper quartile fat cell size values. For univariable and multivariable analyses of continuous variables, data were transformed by the natural log to generate normal distributions. Univariable analyses of continuous variables were performed using Pearson correlation. Stepwise linear regression was performed using age, BMI, respective fat deposition, and other fat cell sizes as predictive variables (see each model for details). The α level for a variable to enter and leave the model was 0.05. Predictive variable estimates are presented as transformed data. All analyses were performed using Minitab Statistical Software (Minitab 20, USA) and GraphPad Prism (Version 9.2.0, GraphPad Software Inc. USA). *P* < 0.05 was considered significant for all analyses.

## Results

### Characteristics and median fat cell sizes of total post-mortem cases and in female and male cases

Females made up ~31% (*N* = 37) of the cases of the 118 total post-mortem cases analysed (Table 1). The median age of the total cohort was 57 ± 24 y (interquartile range) and the median BMI 27.2 ± 6.9 kg/m^2^. There were no differences in median age, BMI, or epicardial, paracardial, or appendix fat cell sizes between female and male cases (Table 1 & Figure S1). Median epicardial and paracardial fat cell sizes were smaller than appendix fat cells when measured in both male and female cases (Table 1 & Figure S1). There was also no discernible difference in the epicardial fat cell size frequency distributions between male and female post-mortem cases, as was reflected by the near identical median (male cases: 2369 µm^2^; female cases: 2380 µm^2^) and upper (Q3, male cases: 3417 µm^2^; female cases 3498 µm^2^) and lower quartile (Q1, male cases 1541 µm^2^; female cases: 1563 µm^2^) fat cell size values (Figure S2).

**Table 1.** Characteristics and fat cell sizes of total post-mortem cases and in female and male cases

Variable (median ± IQR)	Total (*N* = 118)	Male (*N* = 81)	Female (*N* = 37)	M vs. F *P* value
**Caseinformation**				
Age (years)	57.0 ± 23.5	56.5 ± 22.0	60.0 ± 32.5	0.38
BMI (kg/m^2^)	27.2 ± 6.9	26.6 ± 6.4	27.9 ± 8.1	0.73
**Fat cell sizes**				
Epicardial (x10^3^ µm^2^)	2.8 ± 1.9	2.8 ± 1.8	2.8 ± 2.1	0.75
Paracardial (x10^3^ µm^2^)	2.8 ± 2.4	3.1 ± 2.4	2.6 ± 2.5	0.47
Appendix (x10^3^ µm^2^)	5.2 ± 4.4^****####^	5.4 ± 4.1^****####^	4.3 ± 3.3^*##^	0.08
Size ANOVA *P* value	<0.0001	<0.0001	<0.0001	

Due to non-normal data distributions, variables are presented as median values ± interquartile ranges (IQR). Male (M) vs. female (F) differences (i.e. column differences) were assessed using Mann–Whitney test. Differences in median fat cell sizes within groups (i.e. row differences) were assessed using Kruskal-Wallis (ANOVA result as indicated) with Dunn’s multiple comparisons test. *P < 0.001, ****P < 0.0001 vs. group epicardial fat cell size, ^##^*P* < 0.01, ^####^*P* < 0.0001 vs. group paracardial fat cell size.

### Univariable fat cell size predictors in whole post-mortem case cohort and after sex separation

**Figure 1.**
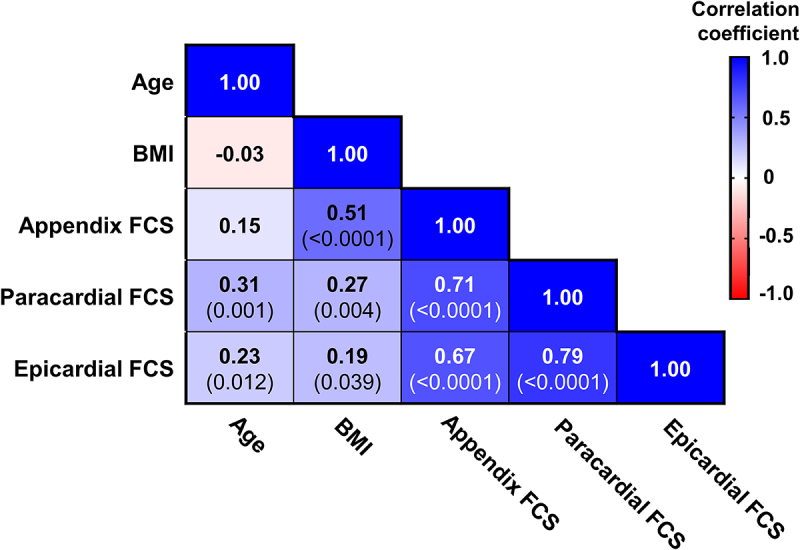
Correlation matrix of information available from all post-mortem cases and fat cell sizes (FCS).

We performed a correlation matrix to identify univariable associations of fat cell sizes (Figure 1). BMI was significantly associated with appendix and paracardial fat cell sizes. Interestingly, BMI was also positively associated with epicardial fat cell size, although the association was weak (*r* = 0.19). Age was positively associated with paracardial and epicardial fat cell sizes, but not with appendix fat cell size. All fat cell sizes were significantly associated with all other fat cell sizes.

**Figure 2.**
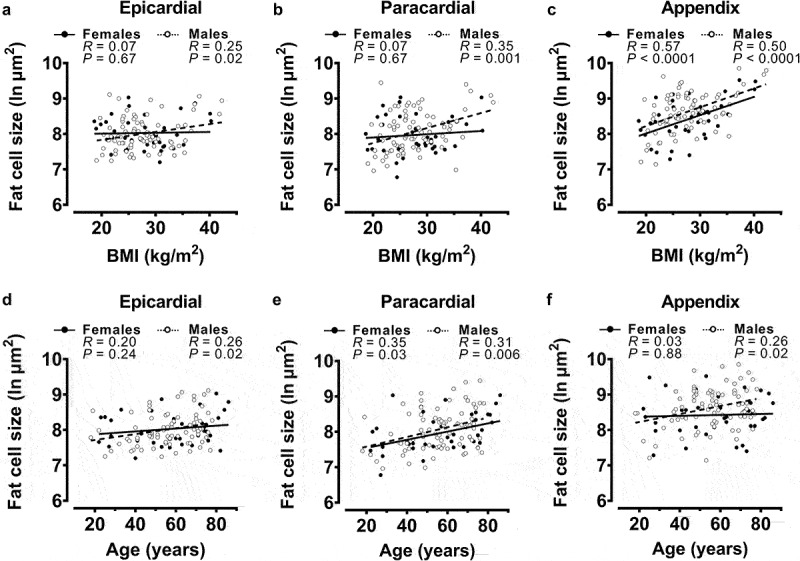
Univariable correlations of fat cell sizes with body mass index (BMI) and age from all post-mortem cases after sex separation.

When separated into male and female post-mortem cases, epicardial fat cell size was significantly and positively associated with BMI (Figure 2a) and age (Figure 2d) in male cases but not in female. A similar sex difference in BMI association was found with paracardial fat cell size (Figure 2b); however, in this depot, age was positively associated in females (Figure 2e) as well as males. Appendix fat cell size was associated with BMI in males (Figure 2c) and females, but only in male cases was there a significant association with age (figure 2f).

Female and male fat cell size correlations with BMI and age were performed using Pearson correlation. Regression line is presented for visual clarity. Relationships between BMI and epicardial, paracardial, and appendix fat cell sizes are shown from **a, b** and **c**, respectively. Sex-dependent associations of epicardial, paracardial, and appendix fat cell sizes with age are shown in **d, e** and **f**, respectively. Fat cell sizes for each adipose depot were natural log (ln) transformed to achieve a normal distribution. *N* = 37 females, *N* = 81 males.

When fat cell size values from male and female cases were sub-divided further into BMI categories, the variations in epicardial (BMI category ANOVA *P* = 0.8; sex ANOVA *P* = 0.9) and paracardial (BMI category ANOVA *P* = 0.6; sex ANOVA *P* = 0.3) fat cell sizes were not significantly associated with obesity status or sex (Figures S3A&B). Conversely, appendix fat cell variation was significantly associated with obesity status when values were grouped in this manner (BMI category ANOVA *P* = 0.001; see Figure for multiple comparisons *P* values); however, no significant relationship with sex was observed (sex ANOVA *P* = 0.053) (Figure S3C).

In addition, when male and female cases were grouped into age categories (18–40 y, 41–60 y, 61+ y), no relationship between epicardial fat cell size with age category or sex was observed (age ANOVA *P* = 0.08; sex ANOVA *P* = 0.7) (Figure S3D). As was evident when age was treated as a continuous variable (Figure 2e), paracardial fat cell size variation was significantly associated with age category (age ANOVA *P* = 0.02, see Figure for multiple comparisons *P* values); however, there was no association with sex (sex ANOVA *P* = 0.6), as shown by the significant increases in paracardial fat cell sizes with increasing age category in male cases only (Figure S3E). Like epicardial fat cell size, the relatively weak association of appendix fat cell size with age in male cases (figure 2f) was not evident when age was grouped into categories (age ANOVA *P* = 0.4) (Figure S3F).

Taken together, these data indicate that the relationships of fat cell size with BMI and/or age are sex-dependent and vary according to whether BMI and age are treated as continuous or categorical variables. Epicardial fat cell size in males only is associated with BMI and age when continuous variables, albeit modestly. Paracardial fat cell size is positively associated with age in male and female cases, and with BMI in male cases only. Finally, appendix fat cell size was more robustly associated with BMI in both sexes; however, in only male cases was there a positive association with age.

### Multivariable regression analysis of fat cell size

The independence of fat cell size predictors was then assessed by stepwise linear regression, with fat cell sizes as response variables and BMI, age, and female sex as predictive variables (Table 2). From this model, age and BMI were found to predict epicardial fat cell size independently but weakly when analysed as total cases or as males only (all cases *R*^2^ adjusted = 0.07, males only *R*^2^ adjusted = 0.095). No independent predictors of epicardial fat cell size could be identified in female cases. Paracardial fat cell size was independently associated with BMI and age in the total case cohort (*R*^2^ adjusted = 0.20) and in males only (*R*^2^ adjusted = 0.22), while in female cases, only age was significantly associated (*R*^2^ adjusted = 0.16). In appendix adipose, alongside BMI and age, female sex was independently predictive of smaller fat cell size (*R*^2^ adjusted = 0.30). BMI was independently associated with appendix fat cell size in both males and females; however, in males age was also predictive. These multivariable analyses show that independent predictors of fat cell size vary by sex. Epicardial fat cell size was independently, yet weakly, associated with age and BMI in male cases, while in female cases no predictors of epicardial fat cell size were identified.

**Table 2.** Stepwise linear regression for predictors of fat cell sizes in all cases and after sex separation

Adipose depot	Epicardial		Paracardial		Appendix	
**All cases**						
**Predictor**	***β***	***P***	***β***	***P***	***β***	***P***
BMI	0.015	0.047	0.029	0.001	0.053	<0.0001
Age	0.007	0.009	0.012	<0.0001	0.007	0.011
Female					−0.251	0.009
Adjusted *R*^2^	0.070		0.203		0.290	
**Males**						
**Predictor**	***β***	***P***	***β***	***P***	***β***	***P***
BMI	0.019	0.047	0.038	0.0009	0.050	<0.0001
Age	0.008	0.027	0.012	0.003	0.009	0.013
Adjusted *R*^2^	0.095		0.221		0.284	
**Females**						
**Predictor**	***β***	***P***	***β***	***P***	***β***	***P***
BMI	No predictors				0.052	0.001
Age			0.012	0.008		
Adjusted *R*^2^			0.163		0.242	

Fat cell sizes were natural log transformed to achieve normal data distributions. Parameter estimates (β) are of transformed data. Model for *All cases* in included body mass index (BMI), age (years), female (yes). Model for *Males* and *Females* only included BMI and age. For variable to enter model α = 0.05, for variable to leave model α = 0.05. *N* = 118 post-mortem cases total, *N* = 81 males, *N* = 37 females.

### Subset post-mortem case characteristics and fat deposition predictors

Relative to other adiposity indices, BMI is a weaker predictor of EAT deposition [3]. Therefore, we analysed a subset of post-mortem cases (*N* = 70) from which EAT volume and epicardial fat cell size were available. From these same cases, extra-pericardial fat volume and visceral fat volume were also measured to provide fat deposition measures related to paracardial and appendix fat cells, respectively. There was no difference in age, BMI, any fat cell sizes, or any fat deposition measures between male (*N* = 48) and female (*N* = 22) cases in the subset cohort (Table S1). All measures of fat deposition were positively associated with age (Figure S4). BMI was univariably associated with EAT volume and visceral fat volume, but not with extra-pericardial fat volume. In stepwise linear regression using BMI, age, and female sex as predictive variables, EAT and visceral fat volumes were independently associated with BMI and age (Table S2). Interestingly, extra-pericardial fat volume associated only with age in the multivariable model (Table S2).

### Sex-specific relationships between fat cell size and anatomically related fat deposition

**Figure 3.**
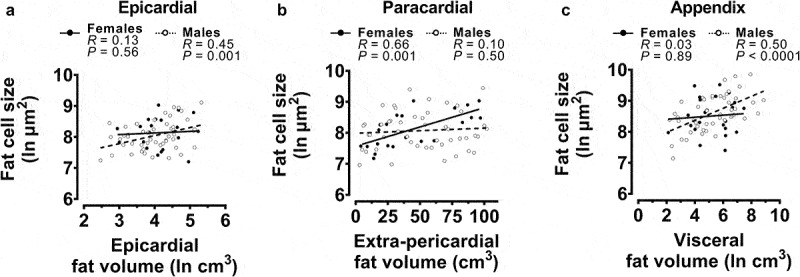
Univariable correlations of fat cell sizes with the related fat volume from the subset of post-mortem cases.

As we found in the whole case cohort, all fat cell sizes were positively associated with BMI, epicardial and paracardial fat cell sizes were associated with age, and all fat cell sizes were associated with all other fat cell sizes (Figure S4). Additionally, the sex-specific relationships with BMI and age found in the whole cohort analysis (Figure 2) were recapitulated in the subset cohort (Figure S5). As with the whole cohort analysis (Figure S3), these sex-specific relationships with BMI and/or age were less clear when each variable was divided into obesity or age categories (Figure S6). Regarding the relationships between fat cell sizes and the anatomically related fat deposition, epicardial, paracardial, and appendix fat cell sizes were positively associated with EAT, extra-pericardial, and visceral fat volumes, respectively (Figure S4). These data suggest that the fat deposition is associated with the size of the constituent fat cells at the univariable level. When separated by sex, univariable analysis found that epicardial (Figure 3a) and appendix (Figure 3c) fat cell sizes positively associated with the deposition of their respective anatomically related fats in males only. Conversely, paracardial fat cell size was significantly related to extra-pericardial fat volume in females only (Figure 3b).

Female and male fat cell size correlations with the anatomically related fat volume/area/thickness were performed using Pearson correlation. Regression line is presented for visual clarity. (**a)** Relationship between epicardial fat cell size with total epicardial fat volume. (**b**) Relationship between paracardial fat cell size with extra-pericardial fat volume. (**c**) Relationship between appendix fat cell size and visceral fat volume. Data were natural log transformed when raw data distribution was non-normal.*N* = 22 females, *N* = 48 males.

To determine the independent associations of BMI, age, sex, and local fat deposition with fat cell sizes, we then performed stepwise linear regression with the inclusion of the anatomically related fat measure (Table 3). EAT volume was the only predictor of epicardial fat cell size when not separated by sex (*R*^2^ adjusted = 0.09) and when grouped into male cases only (*R*^2^ adjusted = 0.16). Despite the addition of the EAT volume, epicardial fat cell size in female cases could not be predicted from the model. Paracardial fat cell size was again positively associated with BMI and age in males (*R*^2^ adjusted = 0.24) and when analysed as total cases (*R*^2^ adjusted = 0.24). In this subset cohort, extra-pericardial fat volume was independently associated with paracardial fat cell size in females (*R*^2^ adjusted = 0.43). Appendix fat cell size was independently predicted by BMI and visceral fat volume in the total subset cohort (*R*^2^ adjusted = 0.29). Only visceral fat volume was associated with appendix fat cell size in males (*R*^2^ adjusted = 0.34) and only BMI was predictive in female cases (*R*^2^ adjusted = 0.20).

**Table 3.** Stepwise linear regression for predictors of fat cell sizes in subset of post-mortem cases with available fat volumes/area

Adipose depot	Epicardial		Paracardial		Appendix	
**All cases**						
**Predictor**	***β***	***P***	***β***	***P***	***β***	***P***
BMI			0.038	0.001	0.038	0.002
Age			0.016	0.0002		
Female						
Total epicardial fat	0.219	0.008				
Extra-pericardial fat						
Visceral fat					0.095	0.032
Adjusted *R*^2^	0.088		0.236		0.288	
**Males**						
**Predictor**	***β***	***P***	***β***	***P***	***β***	***P***
BMI			0.041	0.006		
Age			0.016	0.008		
Total epicardial fat	0.273	0.003				
Extra-pericardial fat						
Visceral fat					0.205	<0.0001
Adjusted *R*^2^	0.161		0.237		0.340	
**Females**						
**Predictor**	***β***	***P***	***β***	***P***	***β***	***P***
BMI	No predictors				0.046	0.021
Age						
Total epicardial fat						
Extra-pericardial fat			0.012	0.001		
Visceral fat						
Adjusted *R*^2^			0.429		0.200	

Fat cell sizes and volumes were natural log transformed according to achieve normal data distribution. Parameter estimates (β) are of transformed data. Model for *All cases* in included body mass index (BMI), age (years), female (yes), and the anatomically related fat deposition measure. Total epicardial fat volume was allocated the depot related to epicardial fat cell size. Extra-pericardial fat volume was allocated the depot related to paracardial fat cell size. Visceral fat volume was allocated the depot related to appendix fat cell size. Model for *Males* and *Females* included variables as above without female (yes). For variable to enter model α = 0.05, for variable to leave model α = 0.05. *N* = 70 post-mortem cases total, *N* = 48 males, *N* = 22 females.

### Relationships of fat cell size in one depot with fat cell size from other depots

Finally, due to the limited accessibility of EAT, it was worthwhile to determine the relationships of epicardial fat cell size with fat cell sizes in other paired fat depots (Table 4). To this end, stepwise linear regression was performed with inclusion of other fat cell sizes in the model, using the whole post-mortem case cohort (*N* = 118). Paracardial fat cell size was independently associated with epicardial fat cell size in all cases (*R*^2^ adjusted = 0.57), in males (*R*^2^ adjusted = 0.67) and in females (*R*^2^ adjusted = 0.43). In all cases and in females only, paracardial fat cell size was also independently associated with age and epicardial and appendix fat cell sizes (*R*^2^ adjusted = 0.72). In male cases, paracardial fat cell size was not associated with age but was independently associated with epicardial and appendix fat cell sizes (*R*^2^ adjusted = 0.77). Alongside BMI and the expected sex-dependency, appendix fat cell size was independently associated with epicardial and paracardial fat cell sizes in the total case cohort (*R*^2^ adjusted = 0.67). In males, appendix fat cell size was associated with BMI and paracardial fat cell size (*R*^2^ adjusted = 0.65), while in female cases epicardial fat cell size was also independently associated (*R*^2^ adjusted = 0.69). These data indicate that fat cell sizes are strongly associated with of other fat cell sizes. Additionally, this exploratory modelling suggests that epicardial fat cell size is associated with paracardial fat cell size in females.

**Table 4.** Stepwise linear regression analysis of fat cell sizes (FCS) as predictors of the size of fat cells in other depots in all cases

Adipose depot	Epicardial		Paracardial		Appendix	
**All cases**						
**Predictor**	***β***	***P***	***β***	***P***	***β***	***P***
BMI					0.034	<0.0001
Age			0.006	0.0007		
Female					−0.187	0.005
Epicardial FCS			0.553	<0.0001	0.251	0.016
Paracardial FCS	0.62	<0.0001			0.508	<0.0001
Appendix FCS			0.425	<0.0001		
Adjusted *R*^2^	0.572		0.720		0.668	
**Males**						
**Predictor**	***β***	***P***	***β***	***P***	***β***	***P***
BMI					0.024	0.002
Age						
Epicardial FCS			0.657	<0.0001		
Paracardial FCS	0.661	<0.0001			0.680	<0.0001
Appendix FCS			0.448	<0.0001		
Adjusted *R*^2^	0.665		0.771		0.652	
**Females**						
**Predictor**	***β***	***P***	***β***	***P***	***β***	***P***
BMI					0.047	<0.0001
Age			0.010	0.002		
Epicardial FCS			0.364	0.026	0.465	0.006
Paracardial FCS	0.600	<0.0001			0.415	0.004
Appendix FCS			0.420	0.001		
Adjusted *R*^2^	0.434		0.630		0.694	

Fat cell sizes were natural log transformed according to achieve a normal distribution. Parameter estimates (β) are of transformed data. Model for *All cases* in included body mass index (BMI), age (years), female (yes), and all other fat cell sizes. Model for *Males* and *Females* included variables as above without female (yes). For variable to enter model α = 0.05, for variable to leave model α = 0.05. *N* = 70 post-mortem cases total, *N* = 48 males, *N* = 22 females.

## Discussion

This study aimed to identify predictors of epicardial fat cell size and to examine whether sex differences exist in epicardial fat cell morphology. In post-mortem cases, we found that epicardial fat cell size is independently associated with age and BMI in males, albeit weakly. Additionally, epicardial fat cell size in males was found to be independently predicted by EAT volume. In contrast, epicardial fat cell size in females could not be predicted by age, BMI, or EAT volume. A similar sex-dependency was found in the predictors of paracardial fat cell morphology, with age and BMI independently associating with paracardial fat cell size in males, while only extra-pericardial fat volume was predictive of paracardial fat cell size in females. These findings provide the first evidence for sex differences in cardiac fat cell morphology and highlight the need to adjust for sex in cardiac adipose research.

### Epicardial fat cell size is differentially predicted in males and females

In general, sex differences in fat cell sizes in adipose depots parallel the patterns of fat accumulation in males and females [9]. For instance, females, who tend to accumulate fat in a gynoid distribution, have been found to have larger subcutaneous gluteal and femoral fat cells relative to BMI- and age-matched males [4,10,11]. Similarly, males, who tend to accumulate fat centrally, also tend to have larger visceral omental fat cells than females [13,14]. This is highlighted in a recent meta-regression analysis showing that the proportion of females included in a study is independently predictive of smaller visceral omental fat cell size [21]. For the current study, we found that female sex was independently predictive of having smaller visceral appendix fat cells, thereby confirming previous findings in central visceral fat cells and validating the use of the fat depot as a paired comparison for epicardial fat cell morphology.

Sex differences in EAT volume or thickness have been reported in previous investigations [15–17]. However, others have found either no sex-dependent difference in total EAT deposition or no univariable association between EAT volume and sex [18,19,22]. These latter studies, instead, found sex differences in the utility of measures of EAT deposition in predicting cardiovascular disease or parameters of cardiac function [16–19,23]. Moreover, differences in EAT thickness or volume have been uncovered if the subject cohort is limited to elderly individuals or when pre- and post-menopausal women were compared [16,17,23,24].

We now show that the median epicardial fat cell size is not different between males and females; however, like some show for total EAT deposition, sex differences do arise in the utility of predictors of epicardial fat cell size. In female cases, epicardial fat cell size was not associated with age, BMI, or EAT volume like that found in male cases. Interestingly, this could suggest that the increased EAT deposition in post-menopausal females is dependent on an increase in fat cell number rather than size [17,23,25]. Our finding of near identical size frequency distributions in male and female epicardial fat cells suggests that a change in cell number does not underlie the sex differences in epicardial fat cell size predictors. Unfortunately, the menopausal status of the female post-mortem cases was not available for this study; therefore, the effect of menopause on epicardial fat cell morphology cannot be properly deduced. It is currently unknown how hormonal shifts during menopause affect epicardial fat biology. However, when considered alongside the differences in EAT deposition found in post-menopausal women, our current findings establish the rationale to more comprehensively investigate the morphology and physiology of epicardial fat cells in females of all ages and all menopausal states. This will be especially relevant in the context of menopause since sex hormone production and sex hormone receptor expression is known to mediate the morphology and physiology of other visceral fat cells [9].

### Identification of independent predictors of epicardial fat cell size

Relative to fat cells from other subcutaneous and visceral fat depots, research of epicardial fat cell morphology has been limited. During our previous investigation of post-mortem cases, we found that epicardial fat cell size is not associated with BMI like that evident in other fat depots [7]. This finding agreed with previous studies in EAT from cardiac surgery patients [26,27] but contrasted with other reports showing positive associations of epicardial fat cell size with BMI in patients with atrial fibrillation or coronary artery disease, or when patients were limited to those with obesity (BMI ≥ 30 kg/m^2^) [5,28,29]. Using a larger sample size, we now find that epicardial fat cell size is associated with BMI at both the univariable level and when combined with age in the multivariable model. Importantly, this association was limited to male post-mortem cases, and the regression model could explain only ~10% of variation in epicardial fat cell size. Therefore, our data suggest that the utility of BMI as a predictor of epicardial fat cell size is limited in comparison to other visceral fat cells.

Considering that EAT constitutes only ~1% of total body fat [1], it is perhaps not surprising that epicardial fat cell morphology is not strongly related to a general adiposity measure like BMI. Hence, we also assessed the relationship between epicardial fat cell size and EAT volume, which should better reflect the local metabolism of EAT. We found that epicardial fat cell size is univariably associated with EAT volume in males, and when EAT volume is added to the regression model it becomes the only independent predictor of epicardial fat cell size. This contrasts with previous reports, including our own, that epicardial fat cell size is not associated with EAT thickness in cardiac surgery patients [8,28]. It does, however, align with a recent study showing a positive univariable association between epicardial fat cell diameter and EAT volume in atrial fibrillation patients [5]. This discrepancy likely arises in the greater resolution of EAT deposition generated by three-dimensional CT imaging and the greater statistical power afforded by a larger sample size. Importantly, the predictive strength of EAT volume for epicardial fat cell size, like that of BMI and age, remains weak in our regression model (*R*^2^ adjusted = 0.16 in males), suggesting that other variables must contribute to regulation of epicardial fat cell morphology. This could include fasting blood glucose, post-prandial insulin, or circulating levels of adiponectin, leptin, inflammatory cytokines, and pre-existing cardiac pathologies, each of which have been associated with epicardial fat cell size [5,26–28].

### Novel predictors of paracardial fat cell size

Alongside the identification of epicardial fat cell morphology predictors, we have also found that paracardial fat cell size is independently associated with BMI and age in male post-mortem cases. This aligns with our previous study, whereby paracardial fat cell size was found to positively correlate with BMI [7]. The current study extends this understanding by showing that age is also associated with paracardial fat cell morphology in males, and that the predictive capacity for each variable is independent. In contrast to male cases, paracardial fat cell size was associated only with age in females. Additionally, when also included in the model, extra-pericardial fat volume was found to independently predict ~43% of the variation in female paracardial fat cell size, suggesting that hypertrophy of paracardial fat cells underlies the expansion of extra-pericardial fat in females. Why this occurs only in females will require further mechanistic investigation and is most likely due to the balance of sex hormones and receptors. Importantly, however, the results of this study establish that a sex difference exists in the predictors of paracardial fat cell size. This study also reinforces the need to avoid conflating the two cardiac adipose tissues by identifying differences in epicardial and paracardial fat cell morphology beyond the biochemical and morphological features described previously [7,30,31].

## Limitations

Our study was most notably limited by information available for each post-mortem case. As a result, other anthropometric indices, such as waist circumference or waist-to-hip ratio, which could function as alternative epicardial fat cell size predictors, were not available for analysis. The same is true for other biochemical and heart function variables that have been proposed to alter visceral fat cell morphology [21]. This would explain the relatively weak *R*^2^ values in our regression models. Despite this lack of case information reducing the pool of testable predictive variables, the lower median age of post-mortem analyses offers a better reflection of the general population than studies of generally older and comorbid cardiac surgery patients.

Further limiting our study was the use of histological analysis to determine fat cell sizes in our post-mortem samples. As noted recently, when compared to other methods, histological analysis yields smaller median fat cell sizes with unimodal, and not bimodal, size distribution curves [32]. This prevents the identification of ‘smaller’ and ‘larger’ fat cell populations and, in turn, the balance of hypertrophic and/or hyperplastic fat cell remodelling. Importantly, fat cell sizes determined from histological analyses of other fat depots have been robustly associated with adiposity measures [32], thereby validating the method for the purposes of our study.

## Conclusion

In conclusion, our study has found that epicardial fat cell size was independently associated with age, BMI, and EAT volume in males, while in females no predictors of epicardial fat cell size could be identified. In addition, we found that BMI and age were independently and positively associated with paracardial fat cell size in males only. In females, instead, paracardial fat cell size was robustly associated with extra-pericardial fat volume. These findings provide the first evidence for sex differences in cardiac fat cell morphology and highlight sex as an important variable in cardiac adipose research.

## Supplementary Material

Supplemental Material

## Data Availability

The data that support the findings of this study are available on request from the corresponding author [HMA]. The data are not publicly available due to containing information that could compromise the privacy of research participants.
