# Supplementary material for: Identifying sex differences in predictors of epicardial fat cell morphology
Source: Adipocyte. 2022 May 19;11(1):325–34. doi: 10.1080/21623945.2022.2073854 (PMC9122305; doi:10.1080/21623945.2022.2073854)
Supplement: Supplemental Material [file KADI_A_2073854_SM2001.docx]

**Supplementary Tables and Figures**

**Identifying sex differences in predictors of epicardial fat cell morphology**

Helen M. M. Waddell^1^, Matthew Moore^2^, Morgan A. Herbert-Olsen^1^, Martin K. Stiles^3,4^, Rexson D. Tse^5^, Sean Coffey^2,6^, Regis R. Lamberts^1^*, Hamish M. Aitken-Buck^1^*

*These authors contributed equally

**Affiliations**

^1^Department of Physiology, HeartOtago, School of Biomedical Sciences, University of Otago, Dunedin, New Zealand.

^2^Department of Medicine, HeartOtago, Dunedin School of Medicine, University of Otago, Dunedin, New Zealand.

^3^Department of Cardiology, Waikato District Health Board, Hamilton, New Zealand.

^4^Waikato Clinical School, University of Auckland, Hamilton, New Zealand.

^5^Department of Forensic Pathology, LabPLUS, Auckland City Hospital, Auckland, New Zealand.

^6^Department of Cardiology, Dunedin Hospital, Southern District Health Board, Dunedin, New Zealand.

| **Table S1. Characteristics, fat cell sizes, and fat volumes/area of subset of post-mortem cases.** | | | | |
| --- | --- | --- | --- | --- |
| **Variable**  (Median ± IQR) | **Total**  (*N* = 70) | **Male**  (*N* = 48) | **Female**  (*N* = 22) | **M vs. F**  **P value** |
| **Case information** | | | | |
| Age (years) | 57.0 ± 23.5 | 56.0 ± 22.0 | 61.0 ± 30.3 | **0.36** |
| BMI (kg/m^2^) | 26.1 ± 8.0 | 26.4 ± 7.1 | 25.3 ± 10.1 | **0.58** |
| **Fat cell sizes** | | | | |
| Epicardial  (x10^3^ µm^2^) | 3.2 ± 2.0 | 3.0 ± 1.8 | 3.5 ± 2.8 | **0.41** |
| Paracardial  (x10^3^ µm^2^) | 3.2 ± 2.9 | 3.0 ± 2.7 | 3.6 ± 3.2 | **0.72** |
| Appendix  (x10^3^ µm^2^) | 5.2 ± 4.9 | 5.3 ± 4.8 | 4.6 ± 5.4 | **0.49** |
| **Fat volumes** | | | | |
| Total epicardial (cm^3^) | 57.9 ± 55.1 | 55.2 ± 58.4 | 60.3 ± 61.3 | **0.72** |
| Extra-pericardial (cm^3^) | 45.0 ± 55.5 | 53.0 ± 50.8 | 34.0 ± 60.8 | **0.36** |
| Visceral (cm^3^) | 28.8 ± 24.4 | 27.7 ± 26.7 | 31.6 ± 20.8 | **0.94** |
| Due to non-normal data distributions, variables are presented as median values ± interquartile ranges (IQR). Male (M) vs. female (F) differences (i.e., column differences) were assessed using Mann-Whitney test. | | | | |

| **Table S2. Stepwise linear regression for predictors of available fat volumes/area.** | | | | | | |
| --- | --- | --- | --- | --- | --- | --- |
| **Adipose depot** | Total epicardial | | Extra-pericardial | | Visceral | |
| **All cases** |  |  |  |  |  |  |
| **Predictor** | ***β*** | ***P*** | ***β*** | ***P*** | ***β*** | ***P*** |
| BMI | 0.065 | <0.0001 |  |  | 0.044 | <0.0001 |
| Age | 0.022 | <0.0001 | 0.876 | <0.0001 | 0.160 | <0.0001 |
| Female |  |  |  |  |  |  |
| Adjusted *R*^2^ | 0.447 | | 0.206 | | 0.417 | |
| Fat volumes/area/thickness were natural log transformed if necessary. Parameter estimates (β) are of transformed data. Model for All cases in included body mass index (BMI), age (years), female (yes). For variable to enter model α = 0.05, for variable to leave model α = 0.05. *N* = 70 post-mortem cases total. | | | | | | |

| **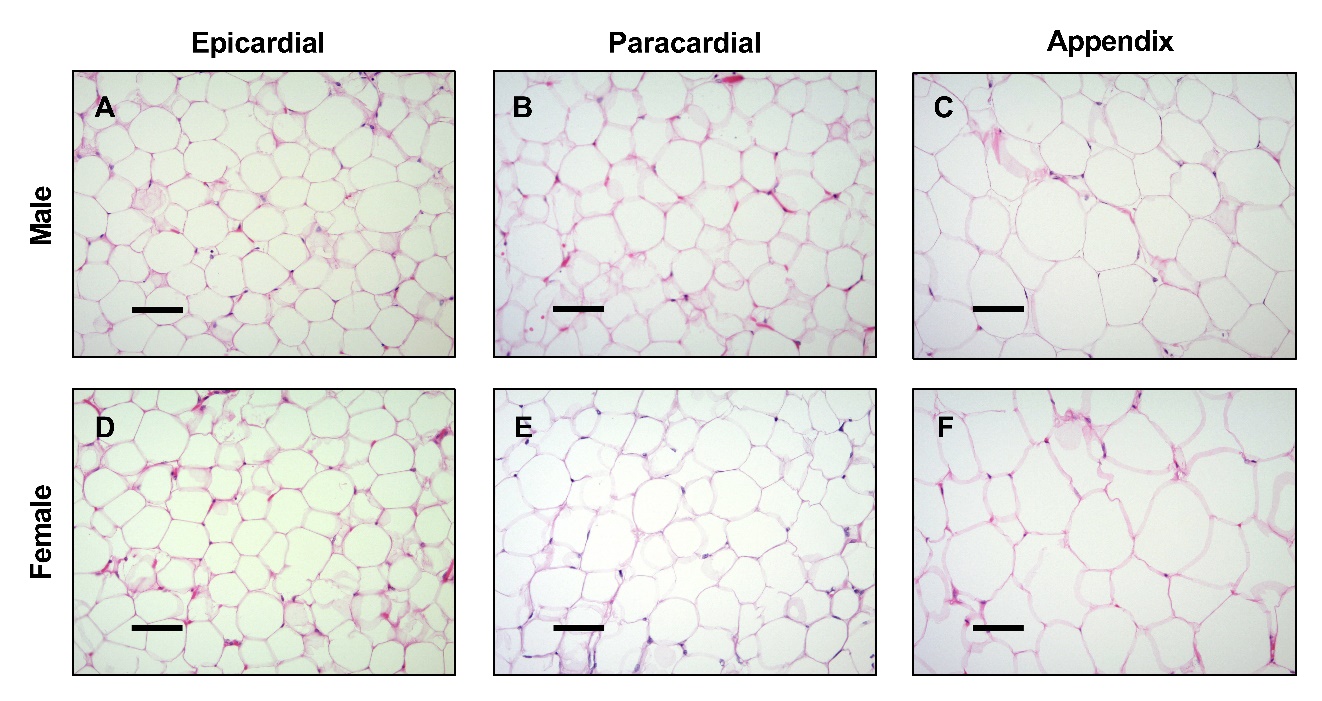** |
| --- |
| **Figure S1. Representative fat section images from male and female post-mortem cases.**  Fat sections were stained with haematoxylin & eosin as part of routine post-mortem examination. Images were acquired at 20x magnification, scale bars represent 100 µm. **A-C**, Representative images of epicardial, paracardial, and appendix fat sections from male cases. **D-F**, Representative images of epicardial, paracardial, and appendix fat sections from female cases. |

|  |  |
| --- | --- |
| **Figure S2. Epicardial fat cell size frequency distributions in male and female post-mortem cases.**  Size frequency distributions were determined for fat cells from epicardial fat of male and female post-mortem cases. Size frequencies are presented as percentage (%) values of total number of fat cells. Fat cell size values are grouped in 500 µm^2^ bins. Median values and lower and upper quartiles are shown in figure. Frequency distributions determined from *n* = 6106 fat cells of *N* = 81 male cases and *n* = 2592 fat cells of *N* = 37 female cases. | |

| 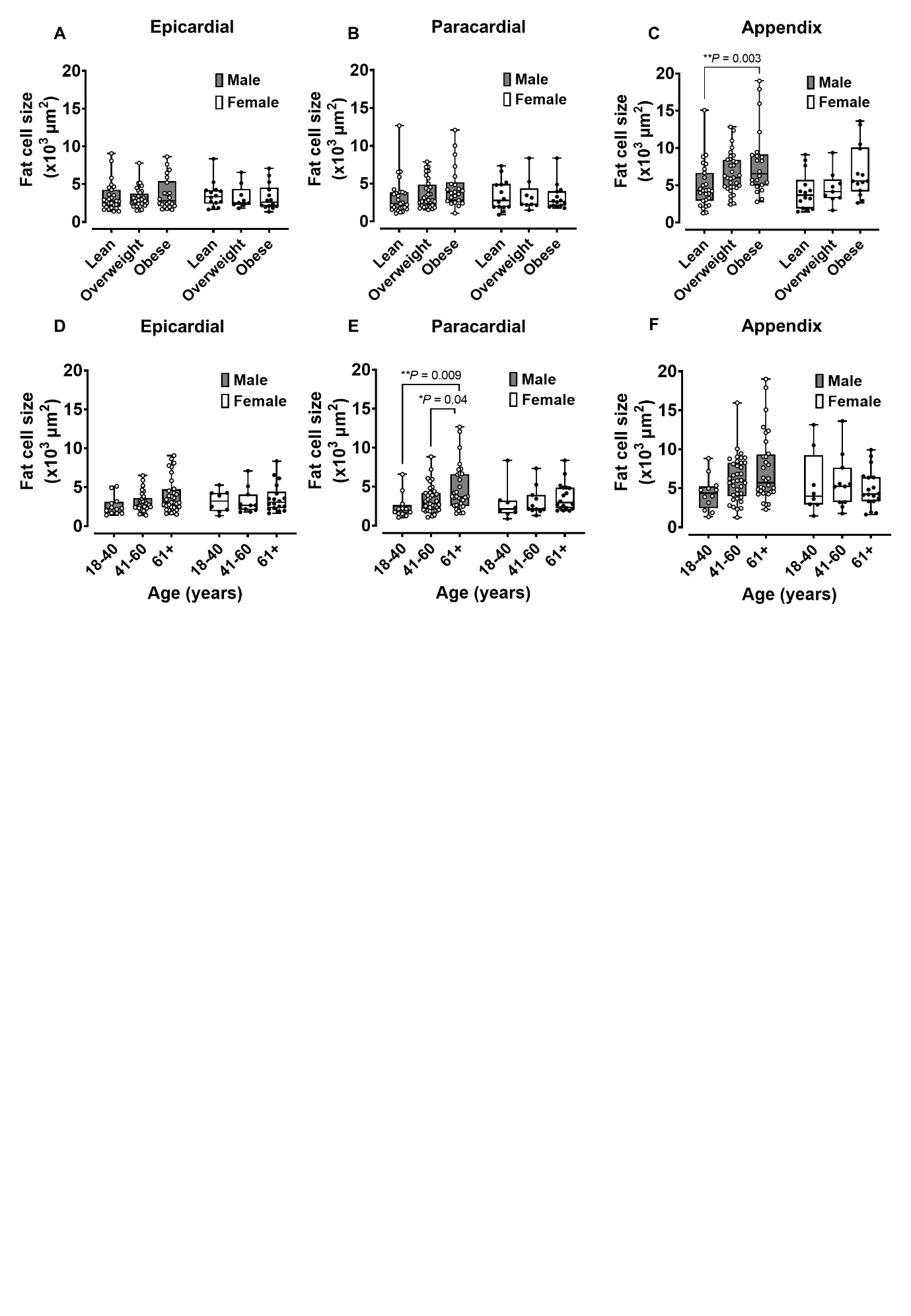 |
| --- |
| **Figure S3. Fat cell size relationships with obesity classification and age category in male and female post-mortem cases.**  **A-C**, Size comparisons of fat cells from epicardial, paracardial, and appendix fat depots in male and female cases stratified by obesity status. Lean: body mass index (BMI) of < 25 kg/m^2^; Overweight: BMI ≥ 25 < 30 kg/m^2^; Obese: BMI ≥ 30 kg/m^2^. **D-F**, Size comparisons of fat cells from epicardial, paracardial, and appendix fat depots in male and female cases stratified by age group. For **A-C**, Male cases: lean *N*=27, overweight *N*=32, obese *N*=22. Female cases: lean *N*=14, overweight *N*=9, obese *N*=14. For **D-F**, Male cases: 18-40 years *N*=12, 41-60 years *N*=37, 61+ years *N*=32. Female cases: 18-40 years *N*=18, 41-60 years *N*=11, 61+ years *N*=18. Raw data were analysed. Differences were determined using two-way ANOVA with obesity status/age group and sex used as factors. Tukey’s multiple comparisons test was performed if significant source of variation was identified by ANOVA. *P* values are as indicated. |

| **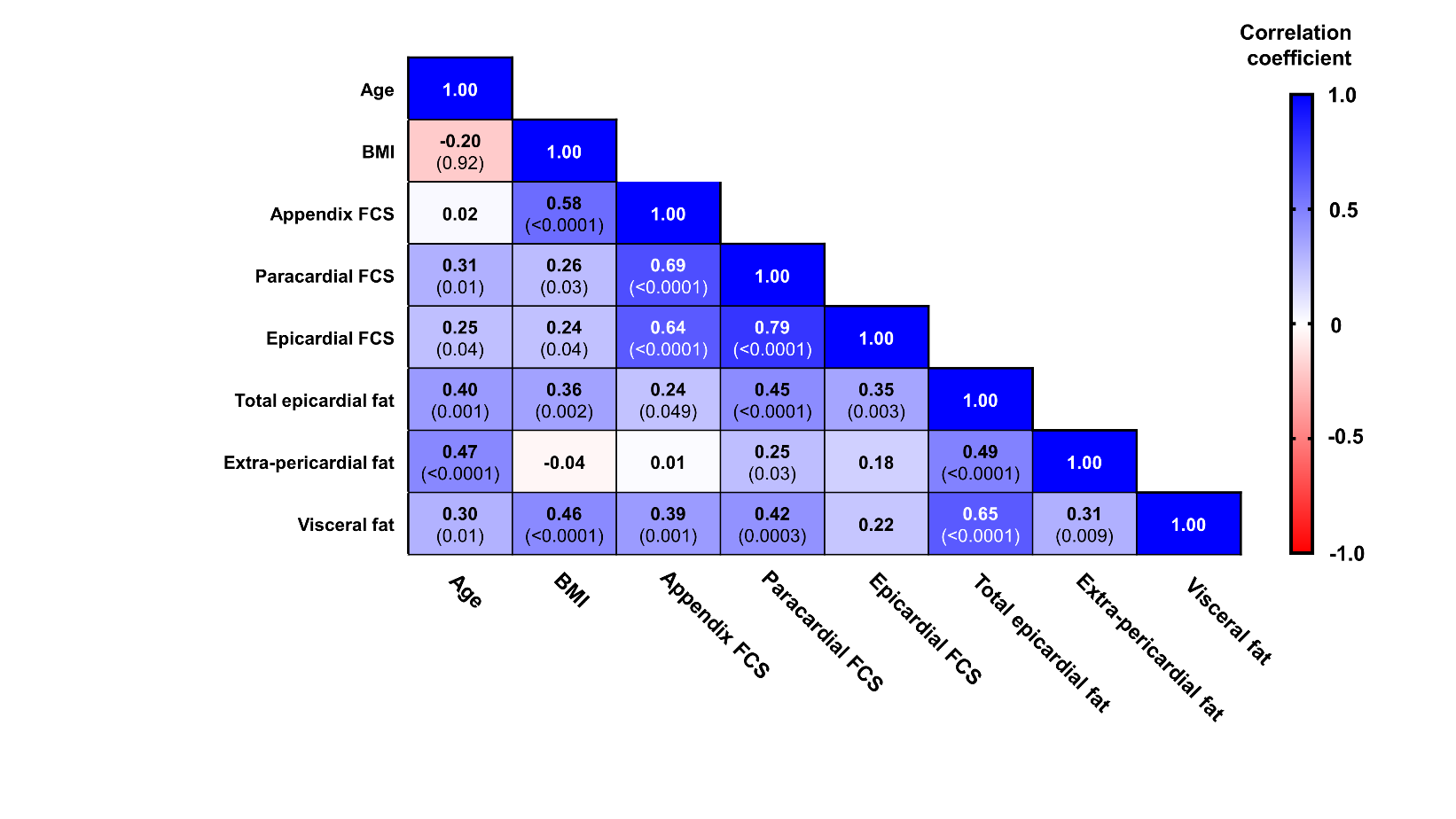** |
| --- |
| **Figure S4. Correlation matrix of information available from the subset of post-mortem cases and fat cell sizes (FCS) and fat volumes.**  Raw data were analysed by Pearson correlation matrix and presented in heat map form. The correlation coefficient (*r*) for each univariate relationship is shown in the figure with the related P value shown in parentheses if *P* < 0.05. Strength of correlation coefficient dictates tile colour, according to scale shown. *N* = 70 post-mortem cases. |
|  |
|  |

| **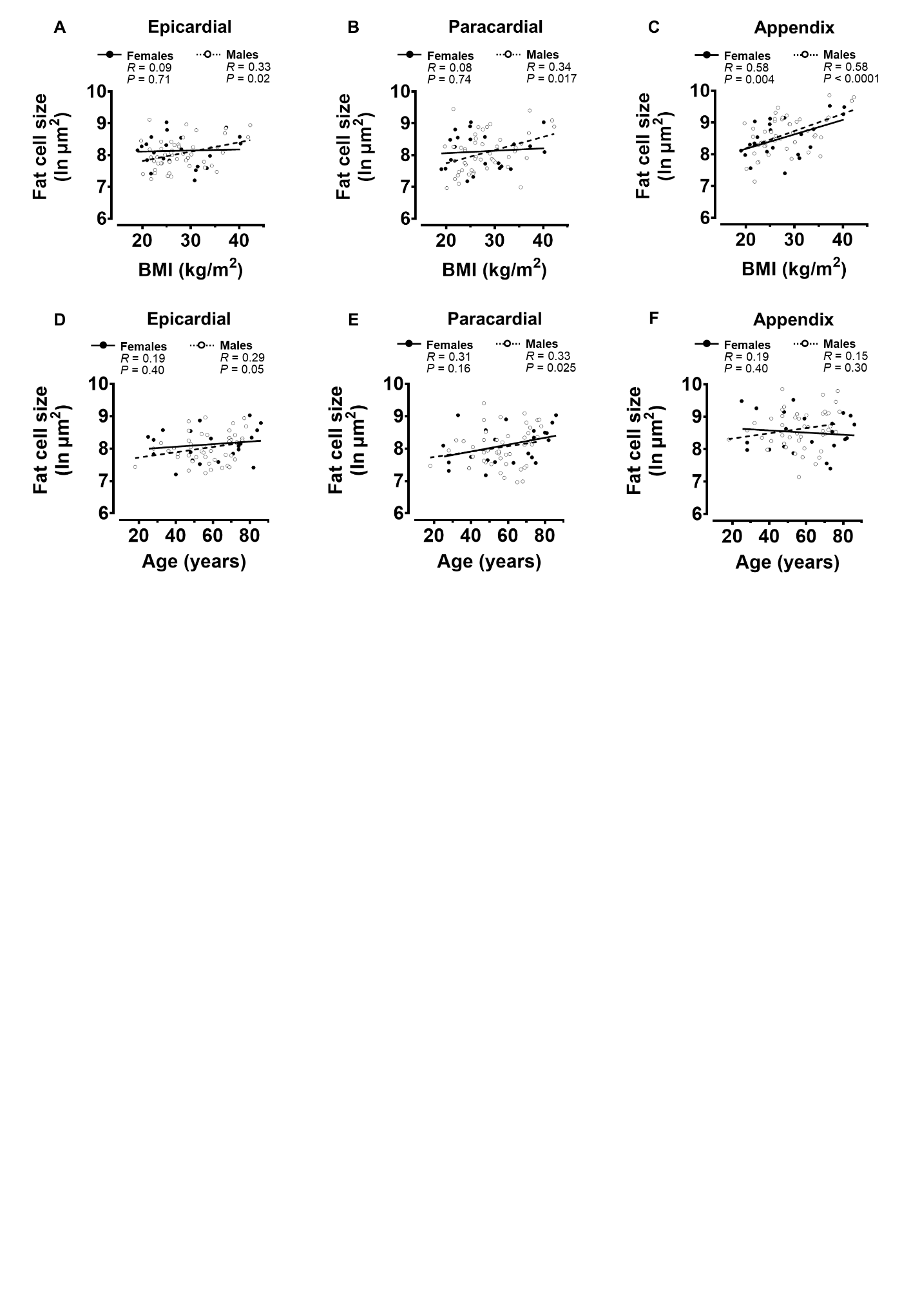** |
| --- |
| **Figure S5. Univariate correlations of fat cell sizes with body mass index (BMI) and age from the subset of post-mortem cases after sex separation.**  Female and male fat cell size correlations with BMI or age were performed using Pearson correlation. Regression line is presented for visual clarity. Relationships between BMI and epicardial, paracardial, and appendix fat cell sizes are shown from **A**, **B**, and **C**, respectively. Sex-dependent associations of epicardial, paracardial, and appendix fat cell sizes with age are shown in **D**, **E**, and **F**, respectively. Data were natural log transformed when raw data distribution was non-normal. *N* = 22 females, *N* = 48 males. |

| **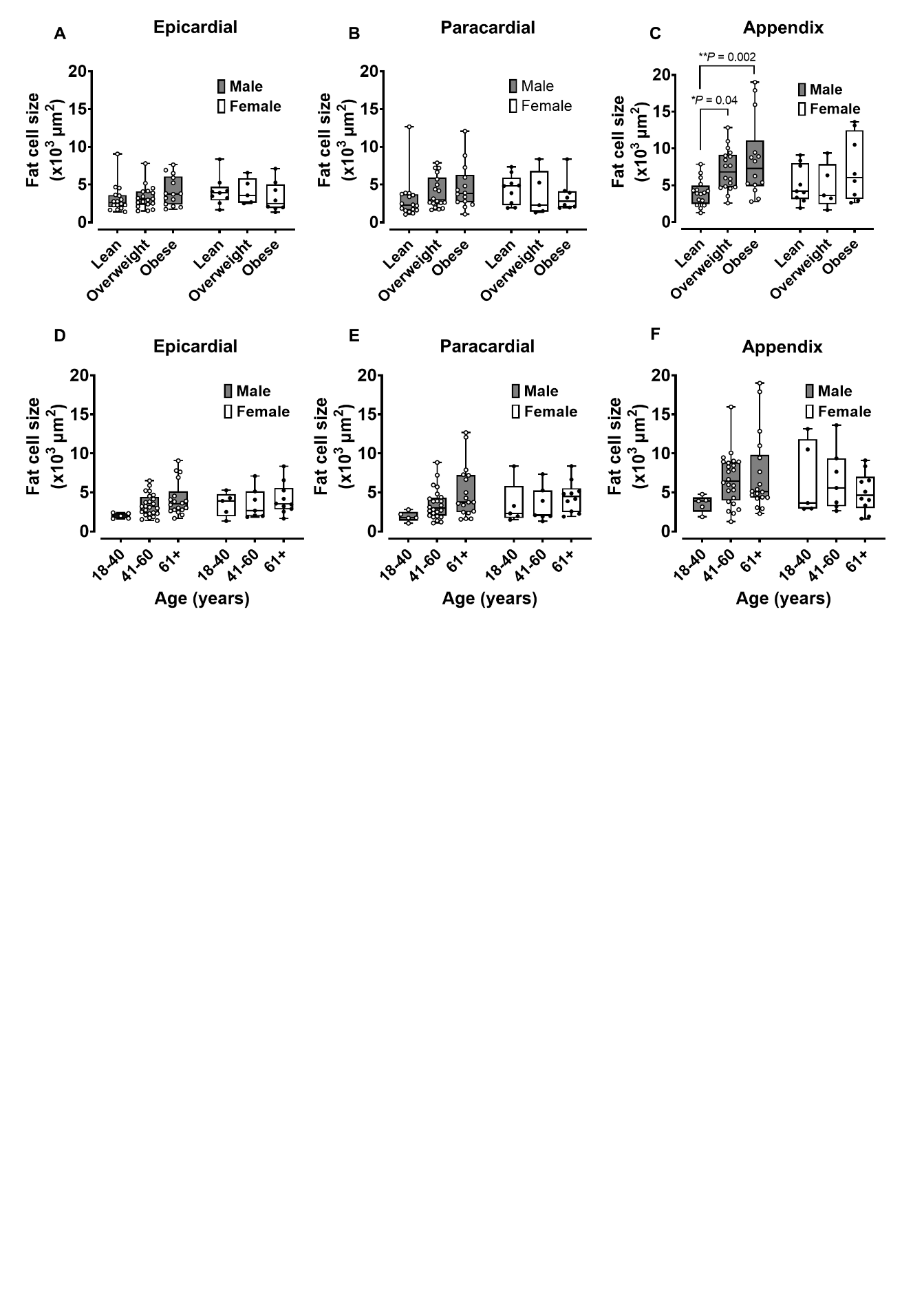** |
| --- |
| **Figure S6. Fat cell size relationships with obesity classification and age category in subset of male and female post-mortem cases.**  **A-C**, Size comparisons of fat cells from epicardial, paracardial, and appendix fat depots in male and female cases stratified by obesity status. Lean: body mass index (BMI) of < 25 kg/m^2^; Overweight: BMI ≥ 25 < 30 kg/m^2^; Obese: BMI ≥ 30 kg/m^2^. **D-F**, Size comparisons of fat cells from epicardial, paracardial, and appendix fat depots in male and female cases stratified by age group. For **A-C**, Male cases: lean *N*=16, overweight *N*=18, obese *N*=14. Female cases: lean *N*=9, overweight *N*=5, obese *N*=8. For **D-F**, Male cases: 18-40 years *N*=5, 41-60 years *N*=24, 61+ years *N*=18. Female cases: 18-40 years *N*=5, 41-60 years *N*=7, 61+ years *N*=10. Raw data were analysed. Differences were determined using two-way ANOVA with obesity status/age group and sex used as factors. Tukey’s multiple comparisons test was performed if significant source of variation was identified by ANOVA. *P* values are as indicated. |
